# Deep Circular RNA Sequencing Provides Insights into the Mechanism Underlying Grass Carp Reovirus Infection

**DOI:** 10.3390/ijms18091977

**Published:** 2017-09-14

**Authors:** Libo He, Aidi Zhang, Lv Xiong, Yongming Li, Rong Huang, Lanjie Liao, Zuoyan Zhu, Yaping Wang

**Affiliations:** 1 State Key Laboratory of Freshwater Ecology and Biotechnology, Institute of Hydrobiology, Chinese Academy of Sciences, Wuhan 430072, China; helibowudi@ihb.ac.cn (L.H.); zhangaidi1010@gmail.com (A.Z.); 18963973548@163.com (L.X.); liym@ihb.ac.cn (Y.L.); huangrong@ihb.ac.cn (R.H.); liaolj@ihb.ac.cn (L.L.); zyzhu@ihb.ac.cn (Z.Z.); 2 University of Chinese Academy of Sciences, Beijing 100049, China

**Keywords:** grass carp, grass carp reovirus, circRNA, differential expression, paternal gene, binding miRNA, blood coagulation, hemorrhagic symptoms, mRNA–miRNA–circRNA network

## Abstract

Grass carp hemorrhagic disease, caused by the grass carp reovirus (GCRV), is a major disease that hampers the development of grass carp aquaculture in China. The mechanism underlying GCRV infection is still largely unknown. Circular RNAs (circRNAs) are important regulators involved in various biological processes. In the present study, grass carp were infected with GCRV, and spleen samples were collected at 0 (control), 1, 3, 5, and 7 days post-infection (dpi). Samples were used to construct and sequence circRNA libraries, and a total of 5052 circRNAs were identified before and after GCRV infection, of which 41 exhibited differential expression compared with controls. Many parental genes of the differentially expressed circRNAs are involved in metal ion binding, protein ubiquitination, enzyme activity, and nucleotide binding. Moreover, 72 binding miRNAs were predicted from the differentially expressed circRNAs, of which eight targeted genes were predicted to be involved in immune responses, blood coagulation, hemostasis, and complement and coagulation cascades. Upregulation of these genes may lead to endothelial and blood cell damage and hemorrhagic symptoms. Our results indicate that an mRNA–miRNA–circRNA network may be present in grass carp infected with GCRV, providing new insight into the mechanism underlying grass carp reovirus infection.

## 1. Introduction

Circular RNAs (circRNAs) are a large class of novel non-coding RNAs with largely unknown functions [1]. Unlike mature messenger RNAs that are linear molecules with distinct 5′ and 3′ termini, the 5′ and 3′ termini of circRNAs are covalently linked and form a closed loop structure [2]. In general, circRNAs are formed by so-called “backsplicing”, in which an upstream splice acceptor is joined to a downstream splice donor [3,4]. In the past, circRNAs were largely considered by-products of mRNA processing linked to mis-splicing [5,6]. However, a recent study found that circRNAs are abundant, widespread, and undergo tissue-specific expression, especially in animals [3,7]. Moreover, some circRNAs are conserved in both sequence and expression patterns, implying a role in cellular functions [8,9]. A number of studies have led to circRNAs being divided into four types: circular exonic RNAs (ecircRNAs), circular intronic RNAs (ciRNAs), exon-intron circRNAs (eiciRNAs), and intergenic circRNAs [2,10,11].

The roles of circRNAs are receiving increasing attention, and they are known to regulate gene expression by acting as miRNA sponges [12,13]. Moreover, circRNAs are associated with disease and may play an important role in pathogenesis and/or diagnosis [14]. For example, Tang et al. (2017) showed that CircRNA_000203 could enhance the expression of fibrosis-associated genes in cardiac fibroblasts [15]. Guo et al. (2016) revealed that circRNA hsa_circ_0000069 is upregulated and promotes cell proliferation, migration, and invasion in colorectal cancer [16]. Chen et al. (2016) suggested that circRNA circPVT1 could be used as a proliferative factor and prognostic marker in gastric cancer [17]. Moreover, other reports suggest a circRNA–miRNA–mRNA network may be present in some diseases [18,19]. However, most of the aforementioned studies focused on humans and other mammals, and research on circRNAs in teleost fish is lacking.

The grass carp (*Ctenopharyngodon idella*), accounting for more than 18% of total freshwater aquaculture production, has been an important aquaculture species in China for over 60 years. The production of grass carp reached 5.5 million tons in 2014, making it the most highly consumed freshwater fish worldwide [20]. Unfortunately, frequent disease outbreaks occur in the grass carp cultivation industry and have caused great economic losses. Specifically, the grass carp reovirus (GCRV), which causes grass carp hemorrhage disease, has received special attention because of the high mortality rate associated with it [21]. Many researches on GCRV have been reported [22,23,24,25,26,27,28], but not many effective drugs or vaccines have been developed until now. Moreover, the host–GCRV interaction is poorly understood, and the mechanism underlying GCRV infection is still unknown.

In this study, grass carp were infected with GCRV, and spleen samples were collected before (control) and after (1, 3, 5, and 7 days) infection. Deep Illumina sequencing was performed to identify the circRNAs involved in host–GCRV interactions and the processing of GCRV infection. Moreover, circRNAs that were differentially expressed before and after GCRV infection were identified, and parental genes and putative binding miRNAs were predicted. The results provide new insight into the mechanism underlying GCRV infection.

## 2. Results

### 2.1. Preliminary Analysis of circRNA Sequencing

At different time points before (control, day 0) and after (1, 3, 5, and 7 days) GCRV infection, spleen tissues from 15 grass carp were collected and used for circRNA sequencing. Three duplicate samples were processed for each time point, yielding a total of 15 libraries that were sequenced on an Illumina Hiseq 2500 platform to generate 150 bp pair-end reads. As shown in Table 1, raw reads, clean reads, a clean base, Q20, Q30, and GC (guanine and cytosine) content for each library were determined. All libraries gave a clean base value ≥9 Gb, Q20 ≥ 94%, Q30 ≥ 86%, and an error rate ≤ 0.05. Therefore, these results proved to have enough depth of the sequencing data and suitability for further study. The sequencing data have been deposited in the Sequence Read Archive (SRA) at the National Center for Biotechnology Information (NCBI) under accession number SRP100592. For the remaining 75 fish, 61 of them died, resulting in a mortality rate of 81.3%. Moreover, the dead fish showed hemorrhagic symptoms, the typical characteristics of GCRV infection (data not shown). Therefore, these results confirmed the efficiency of GCRV infection.

### 2.2. Identification of circRNAs before and after GCRV Infection

**Figure 1:**
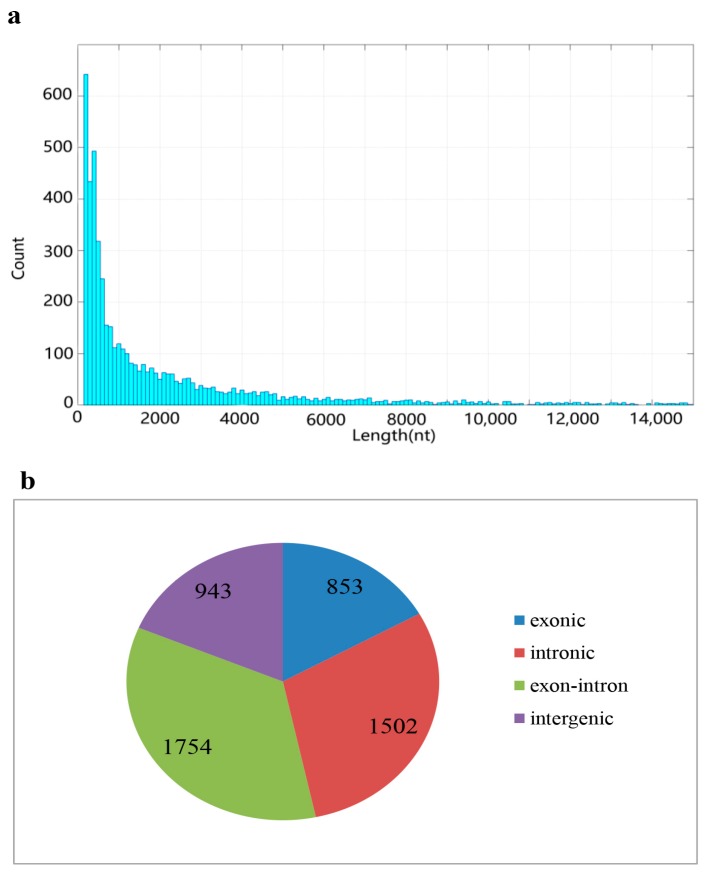
Length and Source statistics of the 5052 circRNAs. (**a**) Length distribution of circRNAs; (**b**) Source statistics of circRNAs.

Clean reads from the 15 libraries were used to identify circRNAs using methods described previously [1]. After calculation and identification, 23,830 candidates were identified, but many were only expressed in one library and the read counts were low. To avoid false positives, other criteria were introduced: (1) only candidates detected in at least three libraries were included; (2) only when the sum of read counts in 15 libraries was greater than 10 were data included. After rigorous selection, 5052 circRNAs were obtained and designated cid_circ_0001 to cid_circ_5052 (Table S1). No circRNAs have been reported in grass carp previously, hence all circRNAs identified were novel. Interestingly, some circRNA isoforms were generated from a single parental gene, suggesting that alternative circularisation may occur in grass carp. A size distribution analysis revealed that the length of circRNAs ranged from 150 to 59,886 bp, but most (~70%) were ≤2000 bp (Figure 1a). Source statistics showed that 16.9%, 29.7%, 34.7%, and 18.7% of circRNAs were exonic, intronic, exon-intron, and intergenic, respectively (Figure 1b). The sequences and junction sequences of all circRNAs are listed in Table S2.

**Figure 2:**
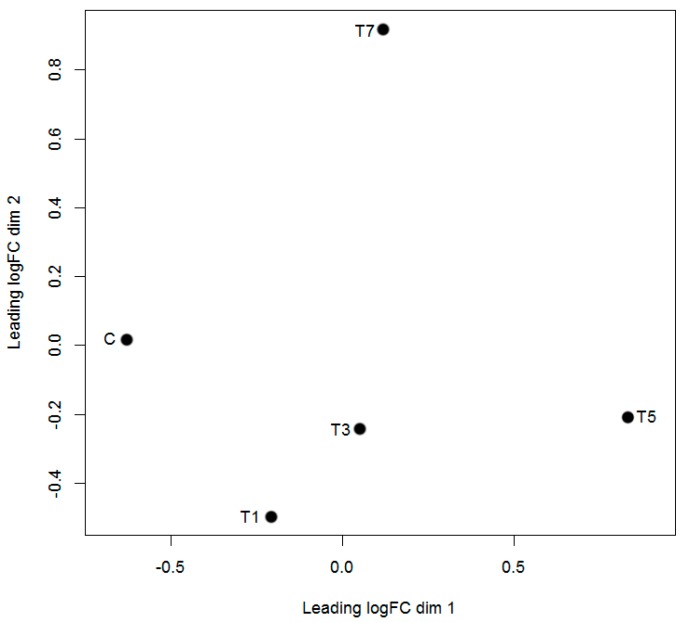
Biological coefficient of variation (BCV) of the five samples. In the plot, dim 1 shows differences between control and infected samples, whereas dim 2 shows differences among infected samples. X axis indicated the coefficient of variation between control sample and infected samples, whereas Y axis represented the coefficient of variation within the infected samples.

We next investigated possible correlations among the five samples. The plotMDS function of the Edge R package was used to produce a plot in which distances between samples corresponded to the leading biological coefficient of variation (BCV) between samples [29]. Control samples were clearly distinct from samples infected with GCRV (Figure 2). Moreover, correlation values increased with increasing time post-infection; the samples from 5 and 7 days post infection (dpi) were not clustered with the samples from 1 and 3 dpi. These results suggested that the efficiency of GCRV infection varied between samples and with time post-infection.

### 2.3. Differentially Expressed circRNAs Following GCRV Infection

**Figure 3:**
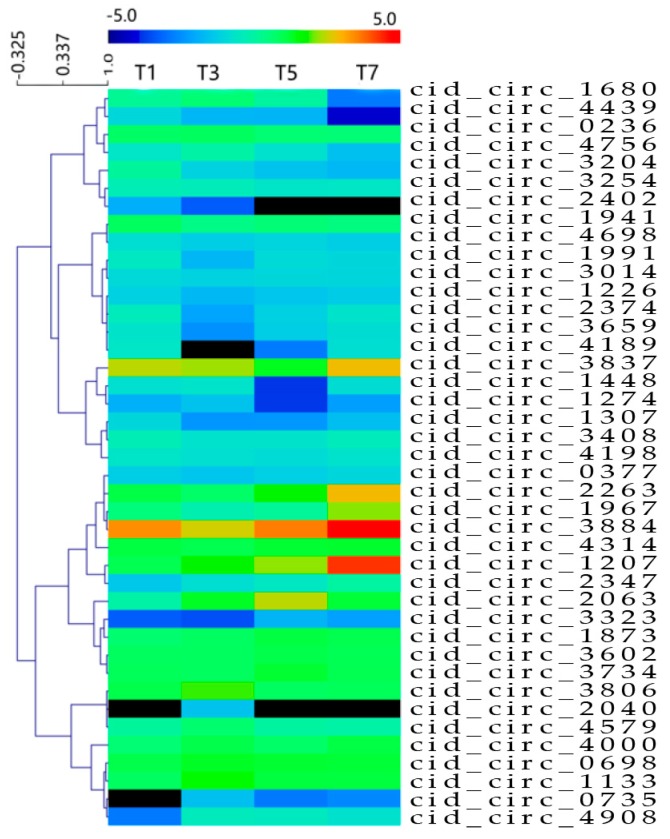
Heatmap of the 41 differentially expressed circRNAs. The heatmap shows log2-fold changes of differentially expressed circRNAs at 1, 3, 5, and 7 days post infection (dpi) compared with controls.

**Figure 4:**
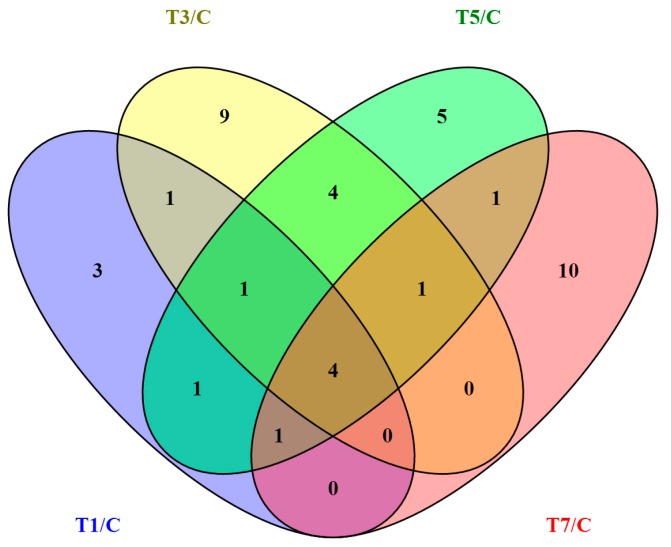
Venn diagram of differentially expressed circRNAs. Differentially expressed circRNAs at 1, 3, 5, and 7 dpi were included in the Venn diagram, and four circRNAs are clearly differentially expressed at all four time points.

To identify circRNAs potentially involved in GCRV infection, expression profiles of circRNAs were examined at 0, 1, 3, 5, and 7 dpi. Uninfected samples were used as controls. As shown in Table 2, 11, 20, 18, and 17 differentially expressed circRNAs were observed at 1, 3, 5, and 7 dpi, respectively (Table S3). The total number of differentially expressed circRNAs at the four time points was 41. A heat map showing the expression patterns of the 41 differentially expressed circRNAs is shown in Figure 3, and a Venn diagram (Figure 4) revealed that four circRNAs were differentially expressed at all four time points.

### 2.4. Characterisation of Parental Genes of Differentially Expressed circRNAs

**Figure 5:**
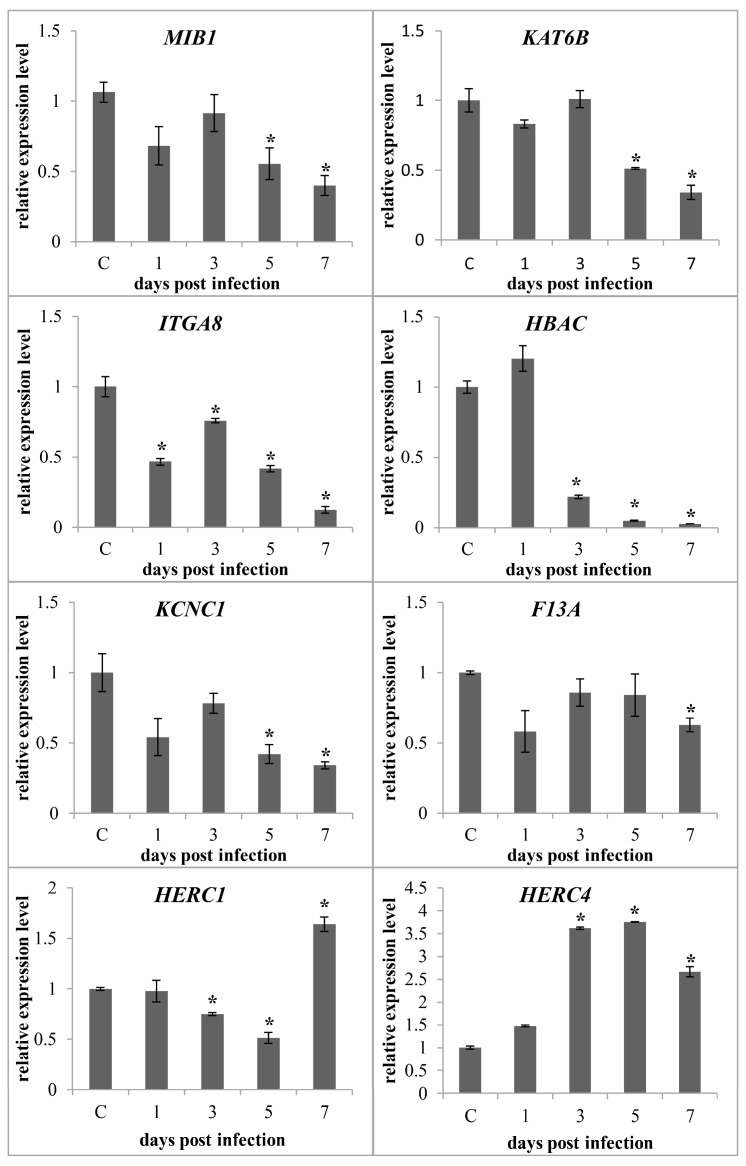
Expression patterns of eight selected parental genes. Eight parental genes were selected for qPCR to validate the expression level at the five time points. The relative expression level of parental genes at different time points were calculated as the ratio of the gene expression level relative to that at 0 dpi (controls). All data are represented as means ± standard deviation of three replicates. Significant differences (*p* < 0.05) between infected and control samples (0 days post-infection) are indicated with an asterisk (*).

Although circRNAs are synthesised via backsplicing and differ from traditional linear mRNA, both are generated from mRNA precursors (pre-mRNA). Thus, characterising the function of linear mRNAs could enhance our understanding of the features of circRNAs. Of the 41 identified differentially expressed circRNAs, 11 were circular intronic RNAs with no parental gene. The remaining 30 were circular exonic RNAs and exon-intron circRNAs originating from 30 parental genes, many involved in metal ion binding, protein ubiquitination, enzyme activity, and nucleotide binding (Table 3). To further investigate the possible roles of parental genes, those involved in metal ion binding and protein ubiquitination were selected for qPCR analysis to investigate their expression patterns following GCRV infection. The primer sequences are listed in Table S4. As shown in Figure 5, of the genes participating in metal ion binding (*MIB1*, *KAT6B*, *ITGA8*, *HBAC*, *KCNC1*, and *F13A*), most were downregulated during the later stages of GCRV infection, whereas genes involved in protein ubiquitination (*HERC1* and *HERC4*) were upregulated during the later stages of infection.

### 2.5. Prediction of Binding miRNAs of Differentially Expressed circRNAs

**Figure 6:**
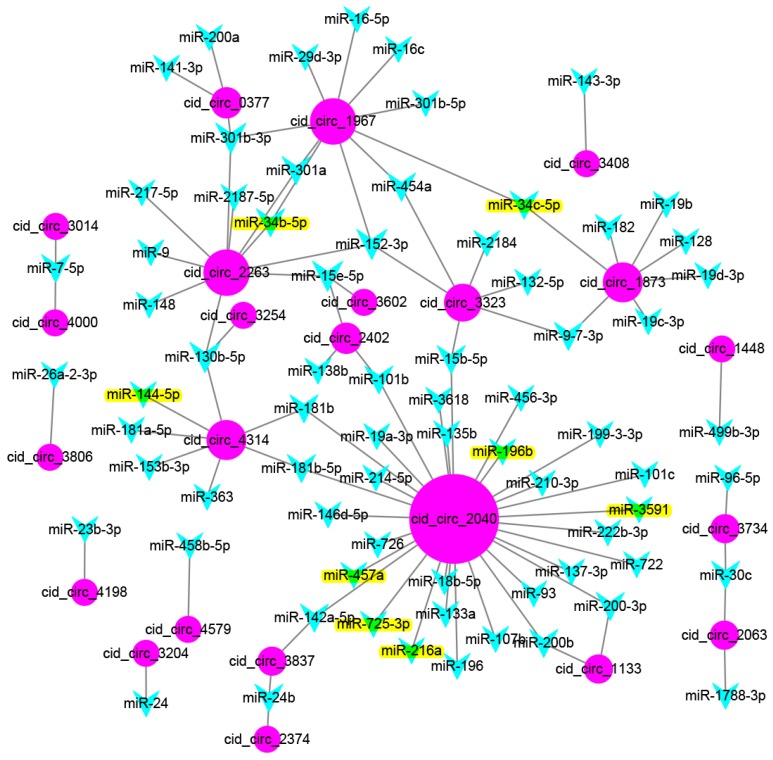
Schematic diagram of interactions between differentially expressed circRNAs and binding miRNAs. MiRanda software was used to predict binding miRNAs for the 41 differentially expressed circRNAs. A total of 1208 grass carp miRNAs was used as a library for target prediction. The results show the network and intersections generated by the software. Circle nodes represent circRNAs, and triangle nodes represent miRNAs. The yellow-stained triangle nodes indicate the differentially expressed miRNAs (miR-144-5p, miR-457a, miR-3591, miR-725-3p, miR-216a, miR-34b-5p, miR-196b, and miR-34c-5p).

Previous studies have suggested that circRNAs could act as miRNA sponges that regulate the expression of corresponding genes at the post-transcriptional level [13,30]. To further investigate the possible role of circRNAs, binding miRNAs of the 41 differentially expressed circRNAs were predicted using MiRanda software. A total of 1208 grass carp miRNAs were used as a library for target prediction [31], which yielded 72 binding miRNAs from the 41 differentially expressed circRNAs (Figure 6). Some circRNAs could bind to several miRNAs, such as cid_circ_2040, which bound to 30 miRNAs. Moreover, some miRNAs could also be bound by several circRNAs. For example, miR-152-3p was bound by three circRNAs. Of the 1208 grass carp miRNAs, 278 corresponded to known miRNAs, and 930 were novel miRNAs. Interestingly, of the 72 binding miRNAs predicted by MiRanda, all were known miRNAs, including miR-7-5p, miR-9, miR-24, miR-34c-5p, and miR-200-3p. In addition, eight binding miRNAs were identified as differentially expressed in our previous study (miR-144-5p, miR-457a, miR-3591, miR-725-3p, miR-216a, miR-34b-5p, miR-196b, and miR-34c-5p) [31].

### 2.6. Confirmation of circRNAs by PCR and RT-qPCR

**Figure 7:**
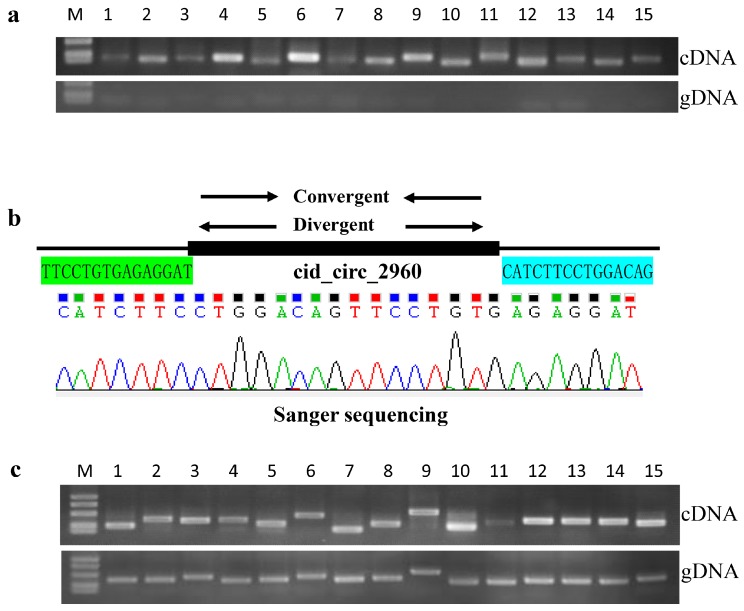
Confirmation of circRNAs by PCR and Sanger sequencing. (**a**) Amplification of circRNAs using divergent primers with cDNA and genomic DNA (gDNA) templates. circRNA forms were only amplified from cDNA samples. M: DNA molecular markers. The sizes of the three bands are 500, 250, and 100 bp (from top to bottom); (**b**) Sanger sequencing confirmation of the head-to-tail backsplicing of circRNA cid_circ_2960. The highlighted (green and blue) nucleotides indicated the junction sequences of circRNA cid_circ_2960; (**c**) Amplification of circRNAs using convergent primers with cDNA and genomic gDNA templates. Convergent primers could amplify the expected bands from both cDNA and genomic DNA samples. M: DNA molecular markers. The sizes of the five bands are 1000, 750, 500, 250, and 100 bp (from top to bottom). Numbers 1–15 refer to cid_circ_0069, cid_circ_0547, cid_circ_1653, cid_circ_3555, cid_circ_4537, cid_circ_3204, cid_circ_1816, cid_circ_2796, cid_circ_3277, cid_circ_4726, cid_circ_0443, cid_circ_1569, cid_circ_2061, cid_circ_2287, and cid_circ_2960.

PCR was carried out to confirm the reliability of the circRNAs obtained using Illumina sequencing. Divergent and convergent primers were designed for each selected circRNA (Table S5). As shown in Figure 7, all 15 selected circRNAs were verified. Specifically, circRNA forms were only amplified by divergent primers in cDNA samples, but not in genomic DNA samples (Figure 7a). Moreover, Sanger sequencing further confirmed the circRNA sequences to be correct (Figure 7b). Meanwhile, the convergent primers could amplify the expected bands in both cDNA and genomic DNA samples (Figure 7c).

**Figure 8:**
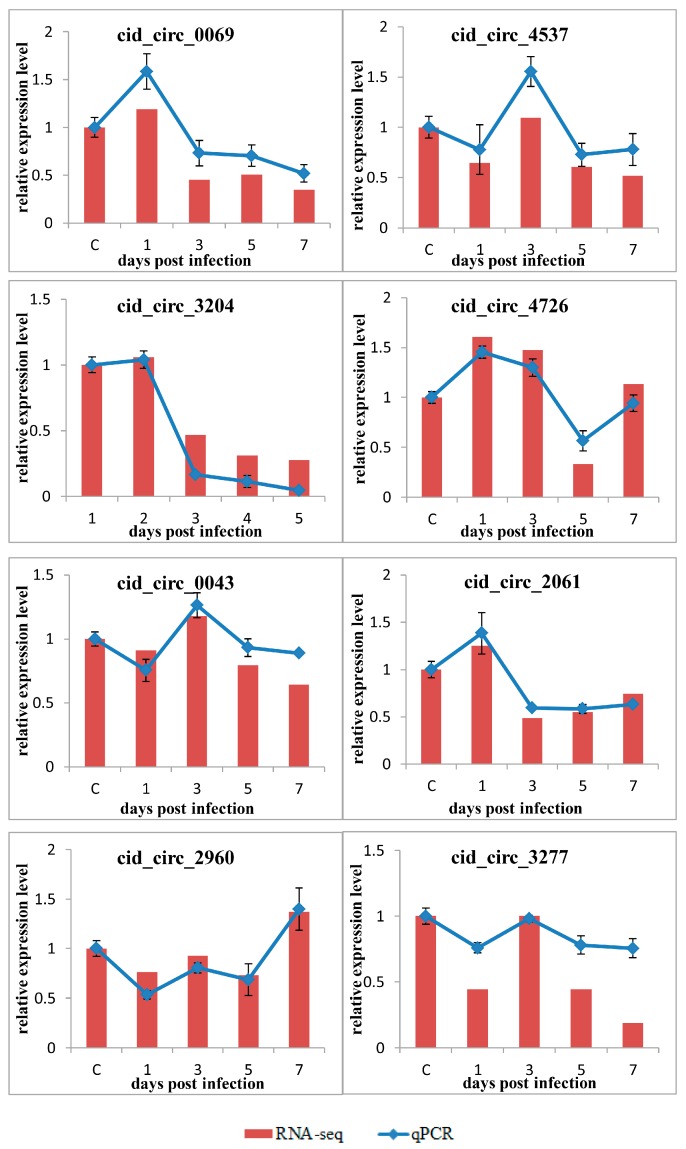
Verification of circRNA expression level by RT-qPCR. Eight circRNAs were randomly selected for RT-qPCR analysis and compared with data obtained using RNA-sequencing (RNA-seq). The relative expression level of circRNAs at different time points were calculated as the ratio of the gene expression level (qPCR) or normalized transcripts per million (TPM) (RNA-seq) relative to that at 0 dpi (controls). The RT-qPCR data are given as means ± standard deviation of three replicates.

To further confirm the expression level of circRNAs, the aforementioned eight circRNAs (cid_circ_0069, cid_circ_4537, cid_circ_3204, cid_circ_4726, cid_circ_0043, cid_circ_2061, cid_circ_2960, and cid_circ_3277) were selected for RT-qPCR using divergent primers. Their relative expression level at different dpi time points was calculated as the amount of expression relative to that measured at 0 dpi. As shown in Figure 8, for most of the examined circRNAs, the expression pattern identified using qPCR was similar to that obtained using an RNA-sequencing (RNA-seq) analysis, although the relative expression level was not always completely consistent. Nevertheless, the results of the qPCR analysis confirmed the reliability and accuracy of the circRNA sequencing data.

## 3. Discussion

In this study, Deep Illumina sequencing and bioinformatics analyses were used to investigate circRNAs in grass carp in response to GCRV infection. Under strict selection, 5052 circRNAs were identified at five time points before and after GCRV infection. Since circRNAs have not been previously reported in grass carp, all circRNAs identified in this study are novel. Interestingly, the number of circRNAs identified was greater than in other species, such as large yellow croaker [32], zebrafish [9], rice [33], and archaea [34]. In our opinion, the large number of circRNAs identified in this study may be due to the deep sequencing of the libraries. Each of the 15 libraries yielded ≥9 Gb of clean data that was sufficiently abundant for further study. It should be noted that many circRNAs were only expressed in some libraries, even though three biological duplicates were performed. This may be an inherent feature of circRNAs, which show temporal and tissue-specific expression pattern differences in many species [3]. Another reason is that the circRNAs with head-to-tail junction reads were randomly sequenced [33]. Thus, the actual number of circRNAs in grass carp may be even more than that reported in the present study.

Although there was no evidence for a relationship between circRNAs and linear forms, understanding the parental genes could help to illuminate the role of circRNAs. Interestingly, many parental genes of the differentially expressed circRNAs are involved in metal ion binding, nucleotide binding, protein ubiquitination, and enzyme activity. qPCR revealed that genes involved in metal ion binding were downregulated following GCRV infection. A previous study suggested that metal ions are important in the immune system’s defence against pathogen invasion [35,36]. Thus, the downregulation of these genes suggests that metal ion homeostasis was disrupted and the immune system was unbalanced. HERC1 and HERC4 are probable E3 ubiquitin-protein ligases involved in ubiquitin-mediated protein degradation [37], and upregulation of these genes during the later stages of infection suggested that protein degradation occurred. However, the relationships between parental genes and their circRNA forms remain tentative and require further investigation.

Increasing evidence suggests that circRNAs act as microRNA sponges that regulate gene expression [30]. To further understand the roles of the circRNAs identified in this study, binding miRNAs among the 41 differentially expressed circRNAs were predicted using MiRanda software, and 72 binding miRNAs were predicted, of which eight were identified as differentially expressed in our previous study [31]. For the eight binding miRNAs, six of them (miR-144-5p, miR-457a, miR-3591, miR-725-3p, miR-196b, and miR-34c-5p) were constantly downregulated during the time points after GCRV infection [31]. Regarding the target genes of the six binding miRNAs, many of them are involved in immune responses, blood coagulation, hemostasis, and complement and coagulation cascades, such as complement component 3 (C3), apolipo A-IV-like (APOA4), inter-α-trypsin inhibitor heavy chain H6-like (ITIH6), signaling lymphocytic activation molecule (SLAM) family member 5 (CD84), and C-X-C chemokine receptor type 4 (CXCR-4) [31]. MiRNAs can interact with specific mRNA targets, resulting in RNA degradation or translational repression [38,39]. Therefore, the six miRNAs that were downregulated may suggest that their target genes upregulated after GCRV infection. Interestingly, we also revealed the overactivity of the complement cascade and immune inflammatory response in grass carp after GCRV infection in our previous study, which is the reason for hemorrhagic symptoms in infected grass carp leading to death [40]. These results therefore suggest that an mRNA–miRNA–circRNA network is present in grass carp following GCRV infection, providing new insight into the mechanisms underlying GCRV.

It should be mentioned that the control group (0 dpi) that was collected before GCRV infection without injecting buffer (without virus) was used for the normalization of transcriptome data with samples that were from the buffer-injected and infected group (1, 3, 5, and 7 dpi). Thus, the transcriptional changes that were found in the infected fish may be due to the intraperitoneal injection effect and not the virus effect. However, the results of circRNAs in the present study are consistent with the results of miRNAs and mRNAs in our previous study. For example, 72 binding miRNAs were predicted for these 41 differentially expressed circRNAs. Eight of them were identified as differentially expressed, and six of them were constantly downregulated after GCRV infection [31]. Many target genes of the differentially expressed miRNAs are involved in immune responses, blood coagulation, hemostasis, and complement and coagulation cascades, which were upregulated after GCRV infection [31]. The overactivity of these genes is the reason for hemorrhagic symptoms in infected grass carp resulting in death [40]. Therefore, the consistency of circRNAs, miRNAs, and mRNAs suggested that the transcriptional changes in the present study were not due to an intraperitoneal injection effect but a virus effect.

In conclusion, 5052 novel circRNAs were identified in grass carp in response to GCRV infection, of which 41 were differentially expressed. Many parental genes of the differentially expressed circRNAs are involved in metal ion binding, protein ubiquitination, nucleotide binding, and enzyme activity. A total of 72 binding miRNAs were predicted for the differentially expressed circRNAs, of which eight were identified as differentially expressed in our previous study, and their target genes are involved in immune responses, blood coagulation, hemostasis, and complement and coagulation cascades. These results provided novel insight into the mechanisms underlying GCRV infection.

## 4. Materials and Methods

### 4.1. Ethics Approval and Consent to Participate

Animal welfare and experimental procedures were carried out according to the Guide for the Care and Use of Laboratory Animals (Ministry of Science and Technology of China, 2006), and the protocol was approved by the committee of the Institute of Hydrobiology, Chinese Academy of Sciences (CAS). The reference number obtained was Y11201-1-301 (Approval date: 30 May, 2016). All surgery was performed under eugenol anaesthesia (Sigma, St. Louis, MO, USA), and all efforts were made to minimise suffering. The eugenol was diluted into water at a concentration of 100 mg/L.

### 4.2. Experimental Fish

Healthy full-sib grass carp without GCRV infection were used at 3 months of age (weight, 3–5 g; average length, 8–10 cm). The fish were randomly selected for GCRV detection by PCR and the results showed that no fish were infected (data not shown). The fish were obtained from the Guan Qiao Experimental Station, Institute of Hydrobiology, Chinese Academy of Sciences, and acclimatized in a circulating water system at 26–28 °C for 1 week before processing. The dissolved oxygen in the water was maintained at 5–10 mg/L, and the photoperiod was 12 h a day. The fish were fed with a commercial diet twice a day (The feed dose of the diet was 1% of the total fish weight every time). The fish were monitored every day, and after no abnormal symptoms (such as lethargy, languishment, and swimming slowly) were observed for 1 week, fish were selected for further experiments.

### 4.3. Virus Challenge and Sample Collection

Dead grass carp with typical hemorrhagic disease symptoms were collected from the field and homogenized together with an equal volume of phosphate buffered solution (PBS). The mixture was centrifuged and then the supernatant was filtered through a 0.22 µm millex filter (Millipore, Billerica, MA, USA). The titer of virus in the supernatant was determined by qRT-PCR (2.97 × 10^3^ copy/µL). The viral suspension was used for further study.

A total of 150 grass carp were used for the virus challenge experiment. These fish were divided into three groups and equally distributed in three tanks (*n* = 50 for each group). Before GCRV infection, 15 fish from the three groups were collected and their spleens were sampled as the uninfected control group (three biological duplicates, *n* = 5 from each group represented a biological duplicate). The remaining fish were infected with 200 µL of GCRV by intraperitoneal injection. At 1, 3, 5, and 7 dpi, 15 fish from the three groups were collected, and their spleens were removed for analysis (three biological duplicates, *n* = 5 from each group represented a biological duplicate). The samples were designated C, T1, T3, T5, and T7 (three biological duplicates for each sample). After sample collection, the remaining fish (75 fish) were used for a mortality calculation in order to investigate the efficiency of GCRV infection.

### 4.4. RNA Isolation, Library Construction, and Sequencing

TRIzol reagent (Invitrogen, Carlsbad, CA, USA) was used to isolate RNA from these samples according to the manufacturer’s protocol. The isolated RNA was further treated by RNase-Free DNase (Promega, Madison, WI, USA) in order to remove possible genomic DNA. RNA concentration and integrity was measured using the Qubit RNA assay kit (Life Technologies, Carlsbad, CA, USA) and the RNA Nano 6000 assay kit (Agilent Technologies, Santa Clara, CA, USA), respectively. High quality RNA was used for further library construction.

RNA (5 µg per sample) was used as input material for RNA sample preparation following the removal of ribosomal RNA using an Epicentre Ribo-zero rRNA Removal Kit (Epicentre, Madison, WI, USA) to obtain rRNA-depleted RNAs. rRNA-depleted RNAs were further treated with RNase R (Epicentre) and subjected to TRIzol extraction. Subsequently, the rRNA-depleted and RNase R-digested RNAs were subjected to sequencing libraries construction by using an NEBNext Ultra Directional RNA Library Prep Kit for Illumina (NEB, Ipswich, MA, USA) following the manufacturer’s protocol. Briefly, the divalent cations under elevated temperature in NEBNext First Strand Synthesis Reaction Buffer were used to fragment the RNA. Random hexamer primer and M-MuLV reverse transcriptase (RNaseH) were used to synthesize the first strand cDNAs. Subsequently, DNA polymerase I and RNase H were prepared to synthesize second strand cDNA. dTTP was replaced by dUTP in the reaction buffer that contained dNTPs. The remaining overhangs were converted into blunt ends via exonuclease/polymerase activities. NEBNext Adaptors with a hairpin loop structure were ligated in preparation for hybridisation after adenylation of the 3′ ends of DNA fragments. Library fragments were purified with an AMPure XP system (Beckman Coulter, Brea, CA, USA) in order to preferentially select cDNA fragments of 150–200 bp in length. In addition, 3 µL of USER Enzyme (NEB, USA) was added with size-selected, adaptor-ligated cDNAs at 37 °C for 15 min followed by 5 min at 95 °C before PCR. Phusion High-Fidelity DNA polymerase, Universal PCR primer, and Index (X) Primer were used to perform the PCR reaction. Finally, the library was purified and qualified by an AMPure XP system and an Agilent Bioanalyzer 2100 system, respectively. Libraries were sequenced on an Illumina Hiseq 2500 platform, and 150 bp paired-end reads were generated. Library construction and sequencing was carried out by Novogene (Novogene, Beijing, China).

### 4.5. Data Analysis

Raw data reads in fastq format were initially processed using in-house perl scripts. In this step, after removing adapters, poly-N sequences, and poor quality data, clean data (clean reads) were obtained. The Q20, Q30, and GC content of the clean data were recorded. All downstream analyses was performed using clean high-quality data.

TopHat2 software [41] was used to map the clean data to the grass carp reference genome [42]. Unmapped reads were kept, and 20-mers from 5′ and 3′ ends were extracted and aligned independently to reference sequences by Bowtie v2.0.6 [43]. Anchor sequences were extended by find_circ1 such that they were completely read-aligned, and the breakpoints were flanked by GU/AG splice sites. Backspliced reads with at least two supporting reads were then annotated as circRNAs. The expression level of circRNAs were normalized as transcripts per million (TPM) using the following formula: normalized expression = (mapped reads)/(total reads) × 1,000,000 [44].

### 4.6. Differential Expression Analysis and Binding miRNA Prediction

Differential expression in two conditions/groups was performed using DESeq2 (version 1.6.3) [45], and *p*-values were adjusted by the Benjamini and Hochberg method. By default, the threshold of corrected *p*-values for differential expression was set to 0.05. MiRanda software was used for binding miRNA prediction of the differentially expressed circRNAs [46], with a threshold of energy score Δ*G* ≤ −16 kcal/mol and paring score *S* ≥ 160. Here, Δ*G* is the free energy of duplex formation that is calculated by a Vienna package from a completely dissociated state, and *S* is the sum of single-residue-pair match scores over the alignment trace.

### 4.7. PCR Amplification and Sanger Sequencing

To confirm the reliability of the data obtained using Illumina sequencing, 15 circRNAs were randomly selected for PCR confirmation. Two sets of primers (divergent and convergent) were designed by Primer Premier 5 software for each selected circRNA (Table S5). In general, the divergent primers were designed near both side sequences of circRNA junctions, and the products’ length ranged from 80 to 150 bp, whereas the convergent primers were the traditional primers for RT-qPCR. The divergent primers were expected to amplify only circRNAs, while the convergent primers could amplify both circRNAs and linear forms. Total RNA was extracted, digested using RNase-Free DNase (Promega) and RNase R (Epicentre), and then purified. A total of 2 µg purified RNA was used to prepare first-strand cDNA by using a random hexamer primer and the ReverTra Ace Kit (Toyobo, Osaka, Japan). Grass carp cDNAs or genomic DNA (20 ng) were used as template for PCR amplification with KOD-Plus-Neo DNA polymerase (Toyobo), and more than 35 cycles were performed. To confirm the PCR results, PCR products of the expected length were subjected to Sanger sequencing by Tsingke Company (Tsingke, Beijing, China).

### 4.8. Validation of circRNA and Parental Gene Expression Level by RT-qPCR

To validate the expression patterns, eight representative parental genes were selected for qPCR using the primers listed in Table S4. cDNAs were obtained as described above, and RT-qPCR was carried out by using fluorescence quantitative PCR instrument (Bio-Rad, Hercules, CA, USA). Each RT-qPCR mixture contained 8.2 µL of ddH_2_O, 10 µL of 2× SYBR Green master mix (Toyobo), 1 µL of template, and 0.4 µL of forward and reverse primer (for each). The *β-actin* gene was used as an internal control for the normalization of gene expression. For each sample, three replicates were included. The program for RT-qPCR was as follows: 95 °C for 10 s, followed by 40 cycles of 95 °C for 15 s, 55 °C for 15 s, and 72 °C for 30 s. Relative expression level was calculated using the 2^−∆∆*C*t^ method [47]. Data are shown as means ± standard deviation of three replicates.

To further confirm the expression level of circRNAs, eight circRNAs were randomly selected from the 15 circRNAs verified in the above RT-qPCR experiments. DNase I- and RNase R-treated RNA was reverse transcribed into cDNA, and RT-qPCR was performed using a fluorescence quantitative PCR instrument (Bio-Rad, Osaka, Japan). Reaction mixtures and the program for RT-qPCR was the same as above, the *β-actin* gene was again used as an internal control for the normalization of gene expression, and the data are expressed as means ± standard deviation of three replicates.

## Figures and Tables

**Table 1:** Summary of circRNA sequencing data.

Sample	Duplicates	Raw Reads	Clean Reads	Clean Bases (Gb)	Error Rate	Q20	Q30	GC Content
C	C-a	70,897,548	61,922,082	9.28	0.03	97.00	92.97	67.28
	C-b	84,758,120	75,871,992	11.38	0.03	97.34	93.48	65.24
	C-c	71,105,834	67,836,022	10.18	0.05	94.06	86.95	65.86
T1	T1-a	70,155,734	65,647,992	9.84	0.04	95.15	89.03	69.19
	T1-b	66,689,052	62,852,844	9.42	0.04	95.16	88.92	67.40
	T1-c	72,041,160	68,785,204	10.32	0.04	95.08	88.81	65.47
T3	T3-a	72,188,758	67,758,712	10.16	0.04	95.17	89.05	67.40
	T3-b	73,758,060	68,452,540	10.26	0.03	96.00	90.70	67.08
	T3-c	70,612,416	66,350,154	9.96	0.03	96.27	91.04	64.72
T5	T5-a	89,647,100	83,873,758	12.58	0.03	96.30	91.03	64.46
	T5-b	79,912,480	74,507,152	11.18	0.03	96.46	91.38	64.51
	T5-c	87,519,928	82,623,624	12.40	0.03	96.49	91.56	64.62
T7	T7-a	67,055,966	62,921,544	9.44	0.03	96.27	91.28	65.49
	T7-b	74,675,616	69,963,696	10.50	0.03	96.55	91.70	64.21
	T7-c	67,217,152	62,582,180	9.38	0.03	96.35	91.35	65.14

**Table 2:** Number of differentially expressed circRNAs following grass carp reovirus (GCRV) infection.

Comparison	Upregulated	Downregulated	Total
T1/C	4	7	11
T3/C	8	12	20
T5/C	7	11	18
T7/C	9	8	17

**Table 3:** Parental genes corresponding to differentially expressed circRNAs.

circRNAs	Parental Genes	Gene Description	Possible Function
cid_circ_0236	*MIB1*	E3 ubiquitin-protein ligase mib1	metal ion binding
cid_circ_0735	*KAT6B*	Histone acetyltransferase KAT6B	metal ion binding
cid_circ_1680	*ITGA8*	Integrin α-8	metal ion binding
cid_circ_1941	*EZH1*	Histone-lysine *N*-methyltransferase EZH1	metal ion binding
cid_circ_2374	*SUCLG2*	Succinyl-CoA ligasesubunit β, mitochondrial (Fragment)	metal ion binding
cid_circ_3014	*NLK*	Serine/threonine-protein kinase NLK	metal ion binding
cid_circ_3204	*HBAC*	Hemoglobin cathodic subunit α	metal ion binding
cid_circ_4439	*KCNC1*	Potassium voltage-gated channel subfamily C member 1	metal ion binding
cid_circ_4698	*F13A*	Coagulation factor XIII A chain	metal ion binding
cid_circ_1307	*HERC1*	Probable E3 ubiquitin-protein ligase HERC1	protein ubiquitination
cid_circ_3884	*HERC4*	Probable E3 ubiquitin-protein ligase HERC4	protein ubiquitination
cid_circ_1448	*UBP45*	Ubiquitin carboxyl-terminal hydrolase 45	protein ubiquitination
cid_circ_4756	*ABTB2*	Ankyrin repeat and BTB/POZ domain-containing protein 2	protein ubiquitination
cid_circ_0377	*AGPAT5*	1-Acyl-sn-glycerol-3-phosphate acyltransferase	enzyme activity
cid_circ_1991	*SPAG9*	C-jun-amino-terminal kinase-interacting protein 4	enzyme activity
cid_circ_3254	*PSTA*	Phosphate transport system permease protein PstA	enzyme activity
cid_circ_3323	*GIMAP2*	GTPase IMAP family member 2	enzyme activity
cid_circ_4000	*SLC12A2*	Solute carrier family 12 member 2	enzyme activity
cid_circ_4579	*SRGAP3*	SLIT-ROBO Rho GTPase-activating protein 3	enzyme activity
cid_circ_1226	*CELF1*	CUGBP Elav-like family member 1	nucleotide binding
cid_circ_3806	*MRPL39*	39S ribosomal protein L39, mitochondrial	nucleotide binding
cid_circ_3837	*ELF2*	ETS-related transcription factor Elf-2	nucleotide binding
cid_circ_0698	*ABCD1*	ATP-binding cassette sub-family D member 1	nucleotide-binding
cid_circ_1133	*LAMA5*	Laminin subunit α-5	other
cid_circ_1967	*TANC2*	Protein TANC2	other
cid_circ_2040	*TMEM187*	Transmembrane protein 187	other
cid_circ_2063	*PHYIP*	Phytanoyl-CoA hydroxylase-interacting protein	other
cid_circ_2263	*GRN*	Granulin-1	other
cid_circ_3408	*DMTN*	Dematin	other
cid_circ_4314	*ASPP1*	Apoptosis-stimulating of p53 protein 1	other

## Supplementary Materials

Supplementary materials can be found at www.mdpi.com/1422-0067/18/9/1977/s1.

## Abbreviations

APOA4	Apolipo A-IV-like
BCV	Biological coefficient of variation
C3	Complement component 3
CD84	SLAM family member 5
circRNAs	Circular RNAs
ciRNAs	Circular intronic RNAs
CXCR-4	C-X-C chemokine receptor type 4
dpi	Days post-infection
ecircRNAs	Circular exonic RNAs
eiciRNAs	Exon-intron circRNAs
F13A	Coagulation factor XIII A chain
GCRV	Grass carp reovirus
HBAC	Hemoglobin cathodic subunit α
HERC1	Probable E3 ubiquitin-protein ligase HERC1
HERC4	Probable E3 ubiquitin-protein ligase HERC4
ITGA8	Integrin α-8
ITIH6	Inter-α-trypsin inhibitor heavy chain H6-like
KAT6B	Histone acetyltransferase KAT6B
KCNC1	Potassium voltage-gated channel subfamily C member 1
MIB1	E3 ubiquitin-protein ligase mib1
PBS	Phosphate buffered solution
Pre-mRNA	mRNA precursors
SLAM	Signaling lymphocytic activation molecule
TPM	Transcripts per million

## References

[1] Memczak S., Jens M., Elefsinioti A., Torti F., Krueger J., Rybak A., Maier L., Mackowiak S.D., Gregersen L.H., Munschauer M. (2013). Circular RNAs are a large class of animal RNAs with regulatory potency. Nature.

[2] Qu S., Yang X., Li X., Wang J., Gao Y., Shang R., Sun W., Dou K., Li H. (2015). Circular RNA: A new star of noncoding RNAs. Cancer Lett..

[3] Jeck W.R., Sorrentino J.A., Wang K., Slevin M.K., Burd C.E., Liu J., Marzluff W.F., Sharpless N.E. (2013). Circular RNAs are abundant, conserved, and associated with ALU repeats. RNA.

[4] Starke S., Jost I., Rossbach O., Schneider T., Schreiner S., Hung L.H., Bindereif A. (2015). Exon circularization requires canonical splice signals. Cell Rep..

[5] Nigro J.M., Cho K.R., Fearon E.R., Kern S.E., Ruppert J.M., Oliner J.D., Kinzler K.W., Vogelstein B. (1991). Scrambled exons. Cell.

[6] Capel B., Swain A., Nicolis S., Hacker A., Walter M., Koopman P., Goodfellow P., Lovell-Badge R. (1993). Circular transcripts of the testis-determining gene *Sry* in adult mouse testis. Cell.

[7] Salzman J., Gawad C., Wang P.L., Lacayo N., Brown P.O. (2012). Circular RNAs are the predominant transcript isoform from hundreds of human genes in diverse cell types. PLoS ONE.

[8] Rybak-Wolf A., Stottmeister C., Glazar P., Jens M., Pino N., Giusti S., Hanan M., Behm M., Bartok O., Ashwal-Fluss R. (2015). Circular RNAs in the mammalian brain are highly abundant, conserved, and dynamically expressed. Mol. Cell.

[9] Shen Y., Guo X., Wang W. (2017). Identification and characterization of circular RNAs in zebrafish. FEBS Lett..

[10] Petkovic S., Müller S. (2015). RNA circularization strategies in vivo and in vitro. Nucleic Acids Res..

[11] Li L., Guo J., Chen Y., Chang C., Xu C. (2017). Comprehensive CircRNA expression profile and selection of key CircRNAs during priming phase of rat liver regeneration. BMC Genom..

[12] Lasda E., Parker R. (2014). Circular RNAs: Diversity of form and function. RNA.

[13] Kulcheski F.R., Christoff A.P., Margis R. (2016). Circular RNAs are miRNA sponges and can be used as a new class ofbiomarker. J. Biotechnol..

[14] Cortés-López M., Miura P. (2016). Emerging functions of circular RNAs. Yale J. Biol. Med..

[15] Tang C.M., Zhang M., Huang L., Hu Z.Q., Zhu J.N., Xiao Z., Zhang Z., Lin Q.X., Zheng X.L., Yang M. (2017). CircRNA_000203 enhances the expression of fibrosis-associated genes by derepressing targets of miR-26b-5p, Col1a2 and CTGF, in cardiac fibroblasts. Sci. Rep..

[16] Guo J.N., Li J., Zhu C.L., Feng W.T., Shao J.X., Wan L., Huang M.D., He J.D. (2016). Comprehensive profile of differentially expressed circular RNAs reveals thathsa_circ_0000069 is upregulated and promotes cell proliferation, migration, and invasion in colorectal cancer. OncoTargets Ther..

[17] Chen J., Li Y., Zheng Q., Bao C., He J., Chen B., Lyu D., Zheng B., Xu Y., Long Z. (2017). Circular RNA profile identifies circPVT1 as a proliferative factor and prognostic marker in gastric cancer. Cancer Lett..

[18] Jin X., Feng C.Y., Xiang Z., Chen Y.P., Li Y.M. (2016). CircRNA expression pattern and circRNA-miRNA -mRNA network in the pathogenesis of nonalcoholic steatohepatitis. Oncotarget.

[19] Nan A., Chen L., Zhang N., Liu Z., Yang T., Wang Z., Yang C., Jiang Y. (2017). A novel regulatory network among LncRpa, CircRar1, MiR-671 and apoptotic genes promotes lead-induced neuronal cell apoptosis. Arch. Toxicol..

[20] Food and Agriculture Organization (FAO) (2016). Fishery and Aquaculture Statistics Yearbook.

[21] Rao Y., Su J. (2015). Insights into the antiviral immunity against grass carp (*Ctenopharyngodon idella*) reovirus (GCRV) in grass carp. J. Immunol. Res..

[22] Wang Q., Zeng W., Liu C., Zhang C., Wang Y., Shi C., Wu S. (2012). Complete genome sequence of a reovirus isolated from grass carp, indicating different genotypes of GCRV in China. J. Virol..

[23] Jian J.C., Wang Y., Yan X.Y., Ding Y., Wu Z.H., Lu Y.S. (2013). Molecular cloning and prokaryotic expression of vp5 gene of grass carp reovirus strain GCRV096. Virus Genes.

[24] Zhou Y., Fan Y.D., Zeng L.B., Ma J. (2013). Prokaryotic expression and immunoassay of grass carp reovirus capsid VP6 protein. Acta Virol..

[25] Jing H.L., Zhang L.F., Fang Z.Z., Xu L.P., Zhang M., Wang N., Jiang Y.L., Lin X.M. (2014). Detection of grass carp reovirus (GCRV) with monoclonal antibodies. Arch Virol..

[26] Zeng W., Wang Y., Liang H., Liu C., Song X., Shi C., Wu S., Wang Q. (2014). A one-step duplex rRT-PCR assay for the simultaneous detection of grass carp reovirus genotypes I and II. J. Virol. Methods.

[27] Zhu B., Liu G.L., Gong Y.X., Ling F., Wang G.X. (2015). Protective immunity of grass carp immunized with DNA vaccine encoding the vp7 gene of grass carp reovirus using carbon nanotubes as a carrier molecule. Fish Shellfish Immunol..

[28] Xu D., Song L., Wang H., Xu X., Wang T., Lu L. (2015). Proteomic analysis of cellular protein expression profiles in response to grass carp reovirus infection. Fish Shellfish Immunol..

[29] Ritchie M.E., Phipson B., Wu D., Hu Y., Law C.W., Shi W., Smyth G.K. (2015). limma powers differential expression analyses for RNA-sequencing and microarray studies. Nucleic Acids Res..

[30] Hansen T.B., Jensen T.I., Clausen B.H., Bramsen J.B., Finsen B., Damgaard C.K., Kjems J. (2013). Natural RNA circles function as efficient microRNA sponges. Nature.

[31] He L., Zhang A., Chu P., Li Y., Huang R., Liao L., Zhu Z., Wang Y. (2017). Deep Illumina sequencing reveals conserved and novel microRNAs in grass carpin response to grass carp reovirus infection. BMC Genom..

[32] Xu S., Xiao S., Qiu C., Wang Z. (2017). Transcriptome-wide identification and functional investigation of circular RNA in the teleost large yellow croaker (*Larimichthys crocea*). Mar. Genom..

[33] Lu T., Cui L., Zhou Y., Zhu C., Fan D., Gong H., Zhao Q., Zhou C., Zhao Y., Lu D. (2015). Transcriptome-wide investigation of circular RNAs in rice. RNA.

[34] Danan M., Schwartz S., Edelheit S., Sorek R. (2012). Transcriptome-wide discovery of circular RNAs in Archaea. Nucleic Acids Res..

[35] Subramanian Vignesh K., Deepe G.S. (2016). Immunological orchestration of zinc homeostasis: The battle between host mechanisms and pathogen defenses. Arch. Biochem. Biophys..

[36] Jesse H.E., Roberts I.S., Cavet J.S. (2014). Metal ion homeostasis in Listeria monocytogenes and importance in host-pathogen interactions. Adv. Microb. Physiol..

[37] Hochrainer K., Mayer H., Baranyi U., Binder B., Lipp J., Kroismayr R. (2005). The human HERC family of ubiquitin ligases: Novel members, genomic organization, expression profiling, and evolutionary aspects. Genomics.

[38] Nilsen T.W. (2007). Mechanisms of microRNA-mediated gene regulation in animal cells. Trends Genet..

[39] Pritchard C.C., Cheng H.H., Tewari M. (2012). MicroRNA profiling: Approaches and considerations. Nat. Rev. Genet..

[40] He L., Zhang A., Pei Y., Chu P., Li Y., Huang R., Liao L., Zhu Z., Wang Y. (2017). Differences in responses of grass carp to different types of grass carp reovirus(GCRV) and the mechanism of hemorrhage revealed by transcriptome sequencing. BMC Genom..

[41] Kim D., Pertea G., Trapnell C., Pimentel H., Kelley R., Salzberg S.L. (2013). TopHat2: Accurate alignment of transcriptomes in the presence of insertions, deletions andgene fusions. Genome Biol..

[42] Wang Y., Lu Y., Zhang Y., Ning Z., Li Y., Zhao Q., Lu H., Huang R., Xia X., Feng Q. (2015). The draft genome of the grass carp (*Ctenopharyngodon idellus*) provides insights into its evolution and vegetarian adaptation. Nat. Genet..

[43] Langmead B., Trapnell C., Pop M., Salzberg S.L. (2009). Ultrafast and memory-efficient alignment of short DNA sequences to the human genome. Genome Biol..

[44] Zhou L., Chen J., Li Z., Li X., Hu X., Huang Y., Zhao X., Liang C., Wang Y., Sun L. (2010). Integrated profiling of microRNAs and mRNAs: MicroRNAs located on Xq27.3 associate with clear cell renal cell carcinoma. PLoS ONE.

[45] Love M.I., Huber W., Anders S. (2014). Moderated estimation of fold change and dispersion for RNA-seq data with DESeq2. Genome Biol..

[46] Enright A.J., John B., Gaul U., Tuschl T., Sander C., Marks D.S. (2003). MicroRNA targets in Drosophila. Genome Biol..

[47] Livak K.J., Schmittgen T.D. (2001). Analysis of relative gene expression data using real-time quantitative PCR and the 2(-Delta Delta C (T)) Method. Methods.

