# Supplementary material for: Deep Circular RNA Sequencing Provides Insights into the Mechanism Underlying Grass Carp Reovirus Infection
Source: Int J Mol Sci. 2017 Sep 14;18(9):1977. doi: 10.3390/ijms18091977 (PMC5618626; doi:10.3390/ijms18091977)
Supplement: Supplementary file 1 [file ijms-18-01977-s001.zip › Supplementary files/Table S4 and S5.pdf]

**Table S4. Primer sequences of 8 parental genes for RT-qPCR.**

| order | gene name | primer sequence | efficiency | Product length (bp) |
|---|---|---|---|---|
| 1 | *MIB1* | GTGCTAACCAAGGCAAATGTCG<br>GCCCACCTTTCCAAGGGTTG | 104.0% | 183 |
| 2 | *KAT6B* | AGCCTGTCAAGGGTTTCGGTAG<br>GGCATGGTTTGGGTCTGGTTTA | 103.1% | 236 |
| 3 | *ITGA8* | CATGGGTCCTAGTCCGGTCAAT<br>CCACAGCCTACTCATCACTCTTACA | 92.9% | 244 |
| 4 | *HBAC* | ATGATCTTATTGGTGGAATGAGCG<br>CAGCGAGGGCAGCAGACAG | 101.3% | 192 |
| 5 | *KCNC1* | ACTACGCCGAAAGGATAGGAGC<br>CAATCACAGGAACAGGCATAGCA | 91.0% | 216 |
| 6 | *F13A* | GCACCTACATTCCTGTCTTCCC<br>GGATGCCATAGGGCGTCA | 96.8% | 165 |
| 7 | *HERC1* | TCAAACTACAGGGTGAGACAGAAGAG<br>CTGGTAACAGCCAGAAGAAATGC | 97.1% | 176 |
| 8 | *HERC4* | AAAGGGCGGCACAGTATTCA<br>GACGATGCGACTGATACGAGTG | 91.9% | 165 |
| 9 | *β-actin* | AGCCATCCTTCTTGGGTATG<br>GGTGGGGCGATGATCTTGAT | 99.2% | 211 |

**Table S5. Divergent and convergent primer sequences of 15 selected circRNAs**

| Order | circRNAs ID | Divergent primer (5'-3') | Convergent primer (5'-3') |
|---|---|---|---|
| 1 | cid_circ_0069 | GATGTCCTGATTGTACTGGCAACA<br>CGCTCTGTCCTCAGCTTTGC | GAACCTGATCTCGTTTCTCTTTTG<br>CCCAGCGAGATGAACACAAAC |
| 2 | cid_circ_0547 | GCTGGATCGAATCGCTAGTATGA | GGTGACCATGCTAACTAGGGTTCTG |

| | | | |
|---|---|---|---|
| | | AAGGACACCCTCGTCGAGATG | TAAACGGCCACGTCATGCAC |
| 3 | cid_circ_1653 | TGGTGCTTACAAGTCTTTCGTGC<br>TTTTCCGTTTTTAGCACCTTGTATC | AAGATACAAGGTGCTAAAAACGGAA<br>GGATGAGGAGACTGAGGAGGTAGA |
| 4 | cid_circ_3555 | CCGGTTACGCTCCTATAAGGTTTG<br>GTGATTCAGCCGGTCATGGG | CCGTCAGTTACCCAGTGAATACAAA<br>GTTCGGTCCTCCAGAGGTTTG |
| 5 | cid_circ_4537 | CGACTCCAAATGGCAGAATGTC<br>GCTTCAAAGTCGCCATTGTTG | AAATCTGCGACCCTGGACTCAC<br>TGGACATTCTGCCATTTGGAGT |
| 6 | cid_circ_3204 | AAATAAAGATGCTGACCGTCTACCC<br>AGATTGTGTGCGAGGATCTTGAAG | ATTTGAATGTTGGTAAGGGACGA<br>TATCTGTATTTATCAGCGAGGGCA |
| 7 | cid_circ_1816 | AGACAGACTCCATTCAGGAGAGGTA<br>CCTCTGCCTGTTTTGCATCTCG | AGGCAGAGGGCTTCCTCAATA<br>CGTCAGATACAAGTCTCCGTCCA |
| 8 | cid_circ_2796 | GTTCGAGTCTAAGAAGAAGCGATGA<br>AAAGGAAGCACAGGCCATATCTTT | CCTTCATCCTCCTCATTCCAGC<br>CTTGGCAAAAAGAAACACACGTC |
| 9 | cid_circ_3277 | CGGACCGGTTCTGGAGGTG<br>CCGCCGACGTGACCTTTC | GCTGAGGTCAAGGAGTTCGG<br>AGGAAGTTGGTGCTCTGGGTCT |
| 10 | cid_circ_4726 | GGGCACTGGTCTTCACTCCTAC<br>GGGGCTTTTCCATCTCTGTACTT | CGTGATTTCTGCCCTGGTGTTT<br>GAAGACCAGTGCCCAGCCTAAA |
| 11 | cid_circ_0443 | TCTTTGCTCCATCCGATTCCC<br>GACGGTTTATGAGGTTCTGCCTG | CTCTTTGTGGAGGTGATGTGATTG<br>GCTGGCGTGCTGCTGGATA |
| 12 | cid_circ_1569 | AAGTTCTTGCCCATTTACAACCC<br>GCAACCACCATCTAGCACCTC | CAAATGAGCAGGAGTCCAAGC<br>GAGGAAGTTAGTGCTTTGCGTTTT |
| 13 | cid_circ_2061 | GTACCAGTACACCGCTCCCTCTT<br>GATCTGCACGGACCGCTG | GTGCCCTGAAACAGCAGAAGA<br>CTCCAGCACCAGCGGGG |
| 14 | cid_circ_2287 | TCTTACCTCTCAGAGAATGCTGGAG<br>GCAGTAGAGGGTCTTGAAGTAGCG | CAGGAACAAGCCCAACGAGAA<br>GCTTATGTTGAGGGCGTCTTTG |
| 15 | cid_circ_2960 | TACAACAACGGCAAATACAAGCA<br>CGCATCCTCTCACAGGAACTG | CTGGCTGCGAGAAGAGGGTTT<br>CTGCTGCTTGTATTTGCCGTTG |
